# Interferon-dependent signaling is critical for viral clearance in airway neutrophils

**DOI:** 10.1172/jci.insight.167042

**Published:** 2023-05-22

**Authors:** Camilla Margaroli, Timothy Fram, Nirmal S. Sharma, Siddharth B. Patel, Jennifer Tipper, Sarah W. Robison, Derek W. Russell, Seth D. Fortmann, Mudassir M. Banday, Yixel Soto-Vazquez, Tarek Abdalla, Sawanan Saitornuang, Matthew C. Madison, Sixto M. Leal, Kevin S. Harrod, Nathaniel B. Erdmann, Amit Gaggar

**Affiliations:** 1 Department of Medicine, Division of Pulmonary, Allergy and Critical Care Medicine,; 2 Program in Protease and Matrix Biology,; 3 Department of Pathology, Division of Molecular and Cellular Pathology, and; 4 Department of Medicine, Division of Infectious Diseases, University of Alabama at Birmingham, Birmingham, Alabama, USA.; 5 Department of Medicine, Division of Pulmonary, Allergy, and Critical Care Medicine, Brigham and Women’s Hospital, Boston, Massachusetts, USA.; 6 Department of Anesthesiology and Perioperative Medicine,; 7 Department of Ophthalmology,; 8 Department of Pathology, Division of Laboratory Medicine, and; 9 Lung Health Center and Gregory Fleming James Cystic Fibrosis Research Center, University of Alabama at Birmingham, Birmingham, Alabama, USA.; 10 Birmingham VA Medical Center, Birmingham, Alabama, USA.

**Keywords:** Immunology, Cellular immune response, Innate immunity, Neutrophils

## Abstract

Neutrophilic inflammation characterizes several respiratory viral infections, including COVID-19–related acute respiratory distress syndrome, although its contribution to disease pathogenesis remains poorly understood. Blood and airway immune cells from 52 patients with severe COVID-19 were phenotyped by flow cytometry. Samples and clinical data were collected at 2 separate time points to assess changes during ICU stay. Blockade of type I interferon and interferon-induced protein with tetratricopeptide repeats 3 (IFIT3) signaling was performed in vitro to determine their contribution to viral clearance in A2 neutrophils. We identified 2 neutrophil subpopulations (A1 and A2) in the airway compartment, where loss of the A2 subset correlated with increased viral burden and reduced 30-day survival. A2 neutrophils exhibited a discrete antiviral response with an increased interferon signature. Blockade of type I interferon attenuated viral clearance in A2 neutrophils and downregulated *IFIT3* and key catabolic genes, demonstrating direct antiviral neutrophil function. Knockdown of *IFIT3* in A2 neutrophils led to loss of IRF3 phosphorylation, with consequent reduced viral catabolism, providing the first discrete mechanism to our knowledge of type I interferon signaling in neutrophils. The identification of this neutrophil phenotype and its association with severe COVID-19 outcomes emphasizes its likely importance in other respiratory viral infections and potential for new therapeutic approaches in viral illness.

## Introduction

Viral respiratory infections are a major cause of worldwide morbidity and mortality (1), as recently evidenced by the COVID-19 pandemic. Severe COVID-19 presentations are characterized by respiratory failure and acute respiratory distress syndrome (ARDS) (2). Robust neutrophilic inflammation characterizes several respiratory viral infections (3), including COVID-19–related ARDS (4). An increased number of circulating monocytes and neutrophils have been reported in SARS-CoV-2 infection (5, 6), and studies in early-stage COVID-19 patients identified a high neutrophil-to-lymphocyte ratio as a biomarker for disease progression (4). Differences in myeloid cell activation in blood at the time of hospitalization have been correlated with disease severity (7, 8), with systemic neutrophil activation in blood at the time of hospitalization correlating with the development of the most severe COVID-19 cases (8).

While the adaptive immune response plays a key role in viral immunity, the role of neutrophils in modulating the inflammatory landscape in viral lung disease such as COVID-19 and their contributions to clinical disease heterogeneity remain poorly defined. Neutrophil heterogeneity is increasingly recognized as a critical regulator of inflammatory disorders (9) but remains poorly understood in viral pathogenesis. Neutrophil subpopulations expressing interferon-stimulated genes (ISGs) have been previously reported in the peripheral blood and spleen at homeostasis, during bacterial infections (10) and in the tumor microenvironment (11). More recently, ISG-expressing neutrophils were found in the blood of patients with severe COVID-19 (12, 13), although functional implications of these gene signatures have not been demonstrated. Here, we investigated the presence of these neutrophil subpopulations in the airways of patients with severe COVID-19 and determined the functional impact of type I interferon signaling in airway neutrophils.

## Results

### Airway neutrophil subsets discriminate patient survival.

**Figure 1:**
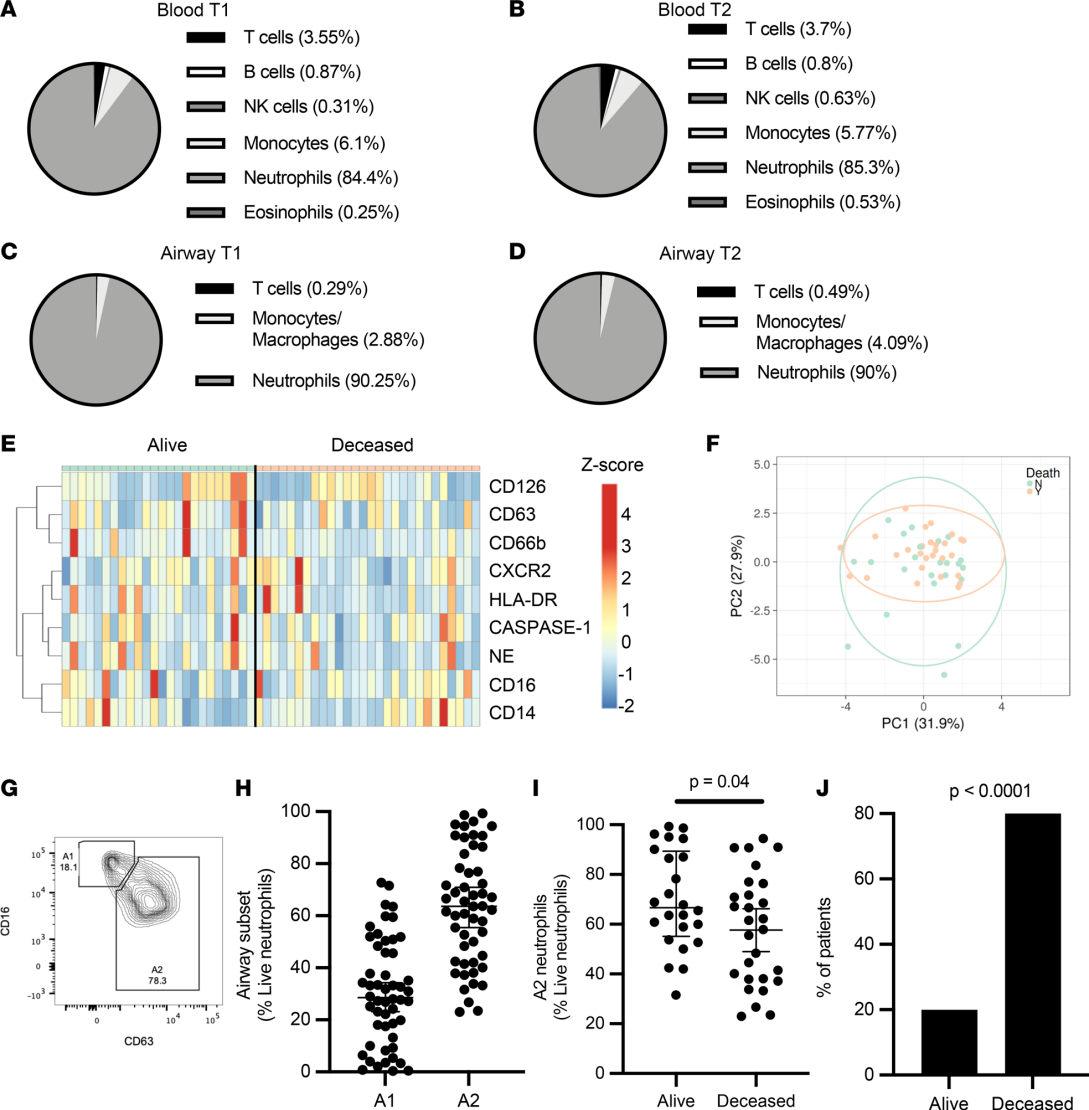
Airway neutrophil subsets associate with survival. Blood and airway immune cell frequencies (live and CD45^+^) and profiles were determined by flow cytometry. COVID-19 patients displayed blood neutrophilia (**A**) upon ICU admission (T1, *n* = 52) (normal neutrophil frequencies: 40%–65%). (**B**) These profiles were maintained at time point 2 (T2, *n* = 28). (**C** and **D**) Airway immune cell frequencies in mBAL fluid displayed marked neutrophil infiltration, which was maintained through both time points. (**E**) No significant difference was observed between surviving and deceased patients for individual surface markers, or as a combined profile by principal component analysis (**F**). (**G** and **H**) Presence of specific neutrophil subsets, including airway neutrophil profiles matching the A1 and A2 populations. (**I**) A2 neutrophil frequency at time of admission discriminated 30-day mortality (alive = 28, deceased = 24). (**J**) Low frequencies of the A2 population correlated with mortality (alive = 3, deceased = 10). Results in **G** and **H** are shown as median and interquartile range. Statistical analysis was performed using an unpaired, 2-tailed *t* test upon normality testing (**I**) and Fisher’s exact test for unpaired analysis (**J**).

We initially investigated innate immune cell frequencies and phenotypes systemically and locally in the lung, and how these profiles changed over time. Blood and airway immune cells from 52 COVID-19 patients requiring intensive care and mechanical ventilation (Supplemental Table 1; supplemental material available online with this article; https://doi.org/10.1172/jci.insight.167042DS1) were collected within 3 days of intubation (14, 15) and patients were resampled again 7 days later (16–19). Analysis by flow cytometry (Supplemental Figure 1) showed that COVID-19 patients displayed marked blood neutrophilia upon ICU admission (compared with normal neutrophil frequencies: 45%–65% of CD45^+^ cells), and low T cell and monocyte frequencies (Figure 1A). No significant differences were observed in blood neutrophils, monocytes, or T cell frequencies over the 2 measured time points (Figure 1B).

Next, we investigated whether neutrophil frequencies in the peripheral circulation were mirrored in the lung. Similar to the systemic profiles, the airway immune cell landscape displayed a predominance of neutrophils that remained consistent over the 2 time points (Figure 1, C and D). Neutrophil frequencies in blood did not statistically correlate with airways at the first time point (Supplemental Figure 2A), but did at time point 2 (Supplemental Figure 2B), and no correlation was found longitudinally between blood at time point 1 and airways at time point 2 (Supplemental Figure 2C). Further, no difference was observed in neutrophil frequencies (systemic or lung) with 30-day survival (28 alive, 24 deceased) at neither time point, nor was there a difference present longitudinally within each group (Supplemental Figure 2D). Neutrophil frequency in recovering patients did not correlate with disease severity, as defined by length of ICU admission (ρ = –0.254), intubation time (ρ = –0.265), hospitalization time (ρ = –0.334), or APACHE II score at time of admission (ρ = –0.455). Likewise, activation profiles of airway neutrophils did not show significant differences over time (Supplemental Figure 2, E–I), and it did not discriminate survival in our 52-patient cohort, either as individual markers (Figure 1E) or in combination by principal component analysis (Figure 1F).

Given the differential neutrophil activation observed in other forms of ARDS (20), we identified the presence of distinct airway neutrophil subsets (A1 and A2), defined by the loss of surface CD16 and release of primary granules as measured by surface CD63 (Figure 1G). These subsets have been previously described in cystic fibrosis airways, with the A2 population entailing phenotypic, metabolic, and transcriptional differences, although the biological role of these cells is unclear (21–23). While A1 and A2 frequencies did not differ over time (Supplemental Figure 2J) and the difference between time point 2 and time point 1 did not discriminate any of the measured clinical parameters, several patients displayed a marked difference in the frequencies of A1 and A2 at time point 1 (Figure 1H), prompting us to investigate whether their frequencies correlated with patient outcomes 30 days after admission to the ICU. Interestingly, ICU patients who survived to 30 days after admission displayed a neutrophil activation profile skewed toward that of the A2 population (Figure 1I). Indeed, lower frequencies of the A2 population (less than 42% of the total neutrophil population) were associated with increased mortality (Figure 1J), suggesting that the A2 neutrophil phenotype may be related to different disease dynamics in COVID-19 patients.

### A2 neutrophils exhibit antiviral transcriptional signatures with increased type I interferon.

**Figure 2:**
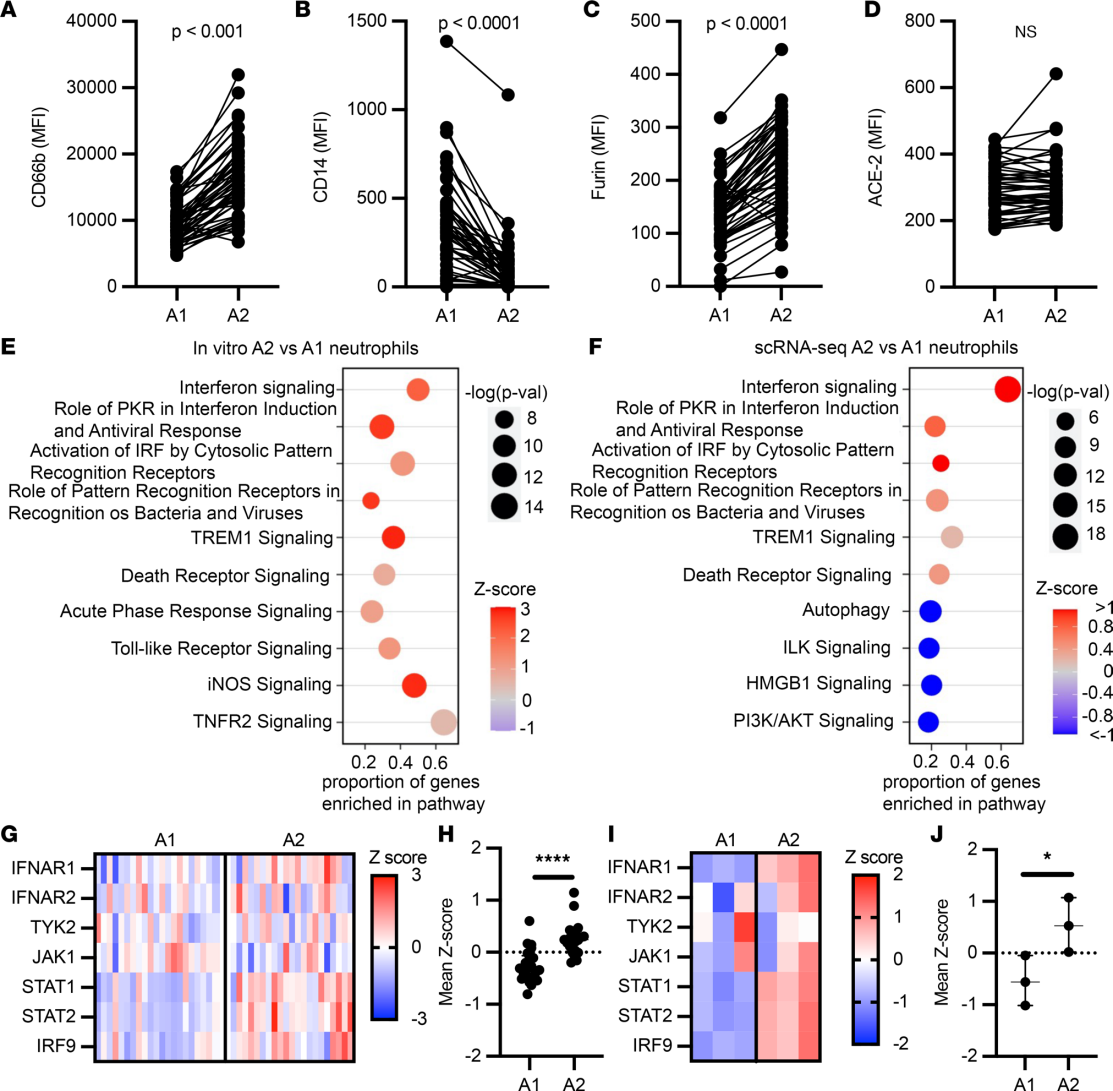
A2 neutrophils show antiviral gene signatures. (**A**–**D**) Airway neutrophils were profiled by flow cytometry at time point 1 (*n* = 52). A1 and A2 neutrophil expression of surface CD66b, CD14, furin, and ACE-2. MFI, median fluorescence intensity. (**E**) Pathway analysis for genes enriched in A2 neutrophils generated in vitro (*n* = 3 donors). (**F**) Pathway analysis for genes enriched in A2 vs. A1 BAL neutrophils from single-cell RNA sequencing (scRNA-seq; *n* = 21 patients). (**G** and **H**) Type I interferon pathway gene expression for A2 vs. A1 BAL neutrophils from scRNA-seq, with mean *z* scores (*n* = 21 patients). (**I** and **J**) Type I interferon pathway gene expression for ex vivo–generated A2 vs. A1 neutrophil mean *z* scores (*n* = 3 per group). Data are shown as median and interquartile range. Statistical analysis was performed using Wilcoxon’s matched-pair signed-rank test for paired analysis (**A**–**D**) and Wilcoxon’s rank-sum test for unpaired analysis (**H** and **J**). *P < 0.05, ****P < 0.0001.

Comparison of neutrophil A1 (CD63^lo^CD16^hi^) and A2 (CD63^hi^CD16^lo^) populations revealed significant differences in the surface expression of activation markers. The A2 population showed increased secondary granule exocytosis (measured as surface CD66b) (Figure 2A), in concordance with the canonical biological mechanisms of neutrophilic granule release, while surface CD14 was significantly higher in the A1 population (Figure 2B). Interestingly, both A1 and A2 neutrophils in COVID-19 patients demonstrated surface expression of furin (Figure 2C) and ACE-2 (Figure 2D), suggesting the potential for interaction with SARS-CoV-2.

Next, given the transcriptional differences previously observed in A1 and A2 neutrophils in other airway diseases, we assessed how the transcriptional profile of A1 and A2 neutrophils relates to their impact on disease severity. To address this, we leveraged an in vitro transmigration model, which has been previously used to generate A1 and A2 neutrophils in the context of other airway diseases (23–25). Here, to better mimic the lung microenvironment of ARDS, we used the cell- and bacteria-free airway fluid from nonviral infectious acute lung injury (ALI) patients who presented A2 neutrophils in their airways (Supplemental Figure 3, A and B) to drive the development of A2 with a clinically relevant stimulus. As a control, A1 neutrophils were generated via transmigration to leukotriene B4, which was previously tested against other major neutrophil chemoattractants (23, 24). A1 and A2 neutrophils differentiated in vitro showed similar activation profiles to those analyzed in vivo (Supplemental Figure 3C), as well as distinct gene expression profiles (Supplemental Figure 3, D and E). A1 neutrophils displayed differential activation of inflammatory signaling pathways (Supplemental Figure 3F), including the IL-17 signaling pathway, a major cytokine in neutrophil-driven inflammation and mucosal immunity. In contrast, the A2 subset showed upregulation of antiviral pathways, most notably interferon signaling (Figure 2E).

To assess the validity of the A2 neutrophil antiviral phenotype, we evaluated the single-cell transcriptional data set from 21 patients with severe COVID-19 published by Bost and colleagues (26) (Supplemental Figure 4A). After quality-control filtering, the 21 samples were concatenated and 48,582 cells were recovered in total. Using the transcriptional profiles of in vitro A1 and A2, as well as expression of lineage-specific marker genes, we identified these 2 neutrophil populations in the single-cell data set from patients with severe COVID-19 (Supplemental Figure 4B). Of the cells sequenced by Bost and colleagues, we identified 25,664 neutrophils by expression of lineage-specific genes such as *FCGR3B* and *CXCR2*, of which 20,600 were A1 (genes: *CD177*, *S100A8*, *S100A9*, and *PROK2*) and 5,064 were A2 (genes: *CD274*, *GBP4*, *GBP5*, *P2RY14*, *IFIT2*, *IFIT3*, and *RSAD2*) (Supplemental Figure 4, C and D). In vivo A2 neutrophils exhibited a unique transcriptional profile that closely mirrored in vitro–generated A2 neutrophils (Supplemental Figure 4E), with increased expression of antiviral genes (Supplemental Figure 4F) belonging to many of the same antiviral pathways enriched in vitro (Figure 2F). Furthermore, A2 neutrophils expressed genes involved in type I interferon signaling, both in vivo (Figure 2, G and H) and in vitro (Figure 2, I and J), with mean *z* scores representing comparable upregulation of the type I interferon pathway in A2 neutrophils, suggesting a differential response upon SARS-CoV-2 encounter compared with A1 neutrophils.

### A2 neutrophils clear SARS-CoV-2.

**Figure 3:**
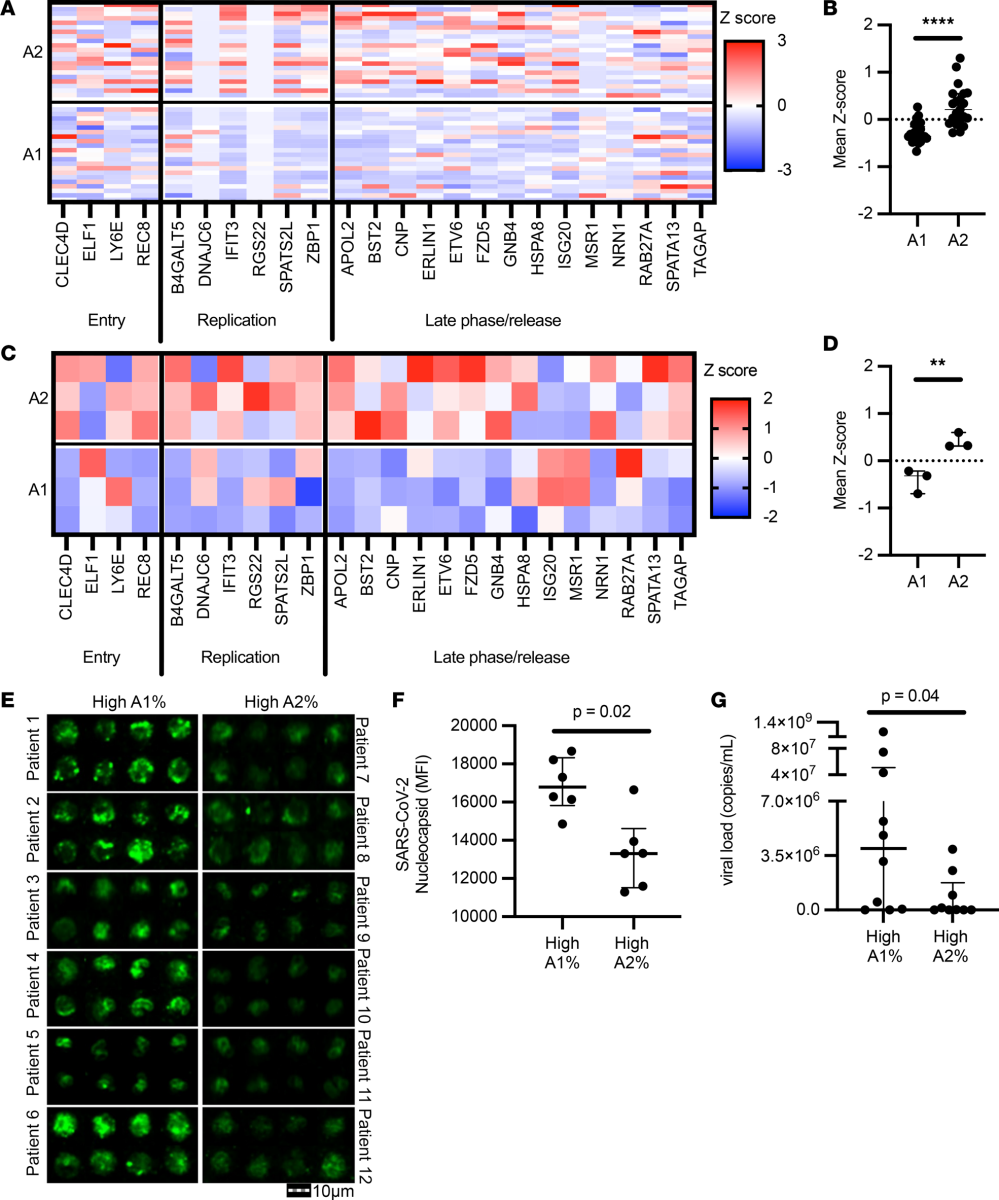
A2 neutrophils show differential anti–SARS-CoV-2 responses. (**A** and **B**) Expression analysis of genes implicated in SARS-CoV-2 intracellular antiviral response, with mean *z* scores. Data were obtained from sc-RNA-seq and each column represents 1 patient (*n* = 21 per group). (**C** and **D**) A1 and A2 neutrophils generated using an in vitro transmigration model showed differential gene expression for SARS-CoV-2 intracellular antiviral response. (**E**) Airway neutrophils from a subset of patients with high A1 or high A2 frequencies (*n* = 6 per group) were stained for SARS-CoV-2 nucleocapsid (green) and acquired by image cytometry (see Supplemental Figure 3). Scale bar: 10 μm. (**F**) Patients with high A1 percentage showed increased presence of intracellular SARS-CoV-2 in airway neutrophils. (**G**) Patients with high A1 percentage showed increased presence of extracellular SARS-CoV-2 in the mBAL supernatant (*n* = 19 patients). Results are shown as median and interquartile range. Statistical analysis was performed using Wilcoxon’s rank-sum test for unpaired analysis. **P < 0.01, ****P < 0.0001.

We next assessed the capacity of A2 neutrophils to modulate SARS-CoV-2 infection. We investigated whether the A2 neutrophils from Bost et al. with the antiviral profile were associated with genes known to have an impact on the SARS-CoV-2 life cycle (27). Indeed, genes involved in the antiviral response to SARS-CoV-2 were upregulated in A2 neutrophils compared with A1 in vivo (Figure 3, A and B). The observed transcriptional changes matched the gene profile of in vitro A2 neutrophils (Figure 3, C and D) and included ISGs, suggesting a functional antiviral role.

To determine whether the transcriptional changes observed in A2 neutrophils were related to alteration of viral loads in vivo, we measured SARS-CoV-2 presence in airway neutrophils using image cytometry (Supplemental Figure 5). Patients with high A1 frequencies had increased intracellular viral staining in airway neutrophils compared with patients with high A2 frequencies (Figure 3, E and F), as well as lower viral copies in the extracellular milieu (Figure 3G), suggesting different disease dynamics when the A2 population is predominant, including differential interaction with SARS-CoV-2 between the 2 neutrophil populations.

**Figure 4:**
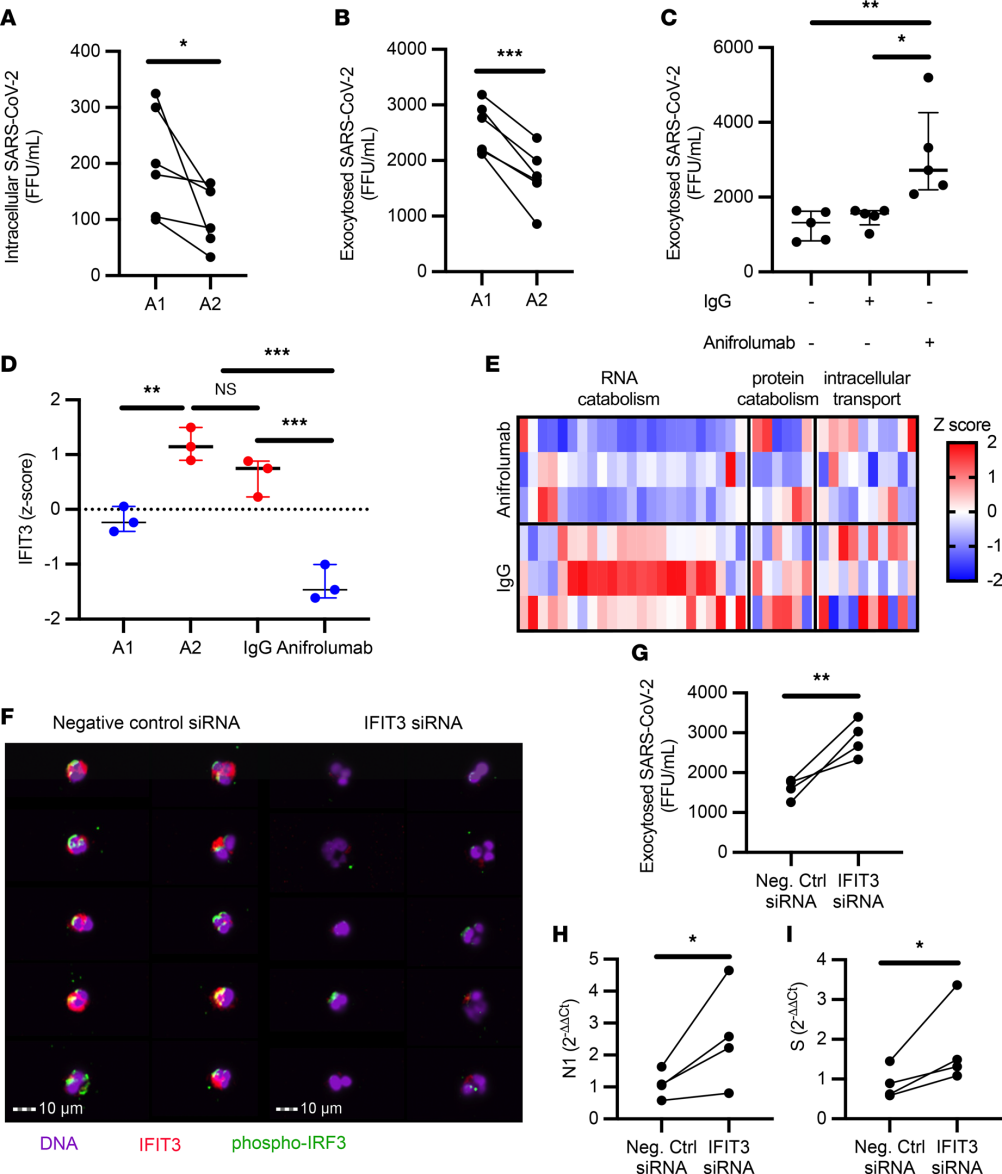
IFIT3 signaling modulates viral clearance in A2 neutrophils. (**A**) A1 and A2 neutrophils incubated with SARS-CoV-2 (MOI = 1) (*n* = 6 neutrophil donors). (**B**) A1 and A2 neutrophils show differential exocytosis of infectious SARS-CoV-2 (*n* = 6 neutrophil donors). (**C**) Type I interferon blockade with anifrolumab increased exocytosis of infectious SARS-CoV-2 in A2 neutrophils (*n* = 5 neutrophil donors) compared with IgG control or media alone. (**D**) *IFIT3* expression by RNA-seq. (**E**) Expression of genes in the macromolecular catabolic processes (GO: 0009057). (**F**) Image cytometry analysis of airway neutrophils for IFIT3 (red) and phospho-IRF3 (green) expression. Nuclei were stained with DAPI (purple). Scale bars: 10 μm. (**G**) *IFIT3* knockdown increased exocytosis of infectious SARS-CoV-2 in A2 neutrophils (*n* = 4 neutrophil donors). (**H** and **I**) *IFIT3* knockdown modulates viral RNA catabolism (*N1* and *S* RNA) in A2 neutrophils (*n* = 4 neutrophil donors). FFU, foci-forming units. Data are shown as median and interquartile range. Statistical analysis was performed using Wilcoxon’s matched-pair signed-rank test (**A**, **B**, and **G**–**I**) or 1-way ANOVA with Tukey’s test for multiple comparisons (**C** and **D**). *P < 0.05; **P < 0.01; ***P < 0.001. NS, not significant.

Next, to address how neutrophils may influence viral dynamics, A1 and A2 populations were generated in vitro and then incubated with SARS-CoV-2 for 1 hour at an MOI of 1, followed by a 24-hour incubation after removal of the extracellular virus. Viral uptake was quantified at 1 hour by measuring extracellular unabsorbed virus. We observed that the uptake of SARS-CoV-2 did not differ between A1 or A2 neutrophil populations (Supplemental Figure 6A), measuring similar amounts of extracellular SARS-CoV-2 in the media of A1 and A2 populations. Further, conditioned media from A1 and A2 populations were incubated directly with virus, resulting in no effect on SARS-CoV-2 infectivity (Supplemental Figure 6B). Likewise, conditioned media placed on VERO cells did not alter the susceptibility of epithelial cells to SARS-CoV-2 infection (Supplemental Figure 6, C and D). Next, we investigated whether A2 neutrophils in vitro had a lower intracellular viral load, as previously observed in vivo by image cytometry. Indeed, we found that A2 neutrophils had reduced amounts of intracellular infectious virus as compared with A1 neutrophils (Figure 4A), in agreement with the observed viral staining by image cytometry (Figure 3C). In both populations, viral replication was low (Supplemental Figure 6E) and there was no detectable difference between the 2 neutrophil subsets. Interestingly, A2 neutrophil populations were also found to have reduced exocytosis of infectious SARS-CoV-2 (Figure 4B), pointing toward differential antiviral functions and viral clearance. Having observed the upregulation of genes in the type I interferon pathway and of ISGs in the A2 population, we blocked type I interferon signaling in A2 neutrophils with a therapeutic monoclonal antibody (anifrolumab) (28) that targets interferon-α/β receptor (IFNAR) and assessed viral clearance by the A2 neutrophil subset. Notably, type I interferon blockade with anifrolumab did not affect A1 neutrophils (Supplemental Figure 6, F–H), while it increased exocytosis of infectious SARS-CoV-2 compared with IgG control or media alone conditions (Figure 4C), showcasing a functional role of the type I interferon pathway in A2 neutrophils. Among the genes known to interfere with SARS-CoV-2, the only one that was significantly affected by the type I interferon blockade was that encoding interferon-induced protein with tetratricopeptide repeats 3 (*IFIT3*, or *ISG60*) (Figure 4D).

IFIT3 has been shown to promote viral clearance in epithelial models of infection through activation of IFN regulatory factor 3–mediated (IRF3-mediated) viral protein and RNA catabolism (29). However the presence and mechanism of action of IFIT3 in airway neutrophils has not been elucidated. To better discern the role of IFIT3 in these cells, we first assessed whether catabolism-associate genes under IRF3 transcriptional regulation (GO: 0009057) were modulated by blockade of type I interferon. Interestingly, treatment with anifrolumab downregulated key IRF3-dependent catabolic genes (Figure 4E). We next determined whether *IFIT3* knockdown would affect these antiviral pathways in A2 neutrophils. Indeed, siRNA knockdown of *IFIT3* (Supplemental Figure 6I) led to loss of IRF3 phosphorylation (Figure 4F), which is required for nuclear translocation and transcription of catabolic genes. Further, while *IFIT3* knockdown did not alter the ability to take up SARS-CoV-2 (Supplemental Figure 6J), it did reduce viral clearance (Figure 4G) through the modulation of viral RNA catabolism (Figure 4, H and I). These results mirrored the alteration of viral clearance obtained upon treatment with anifrolumab. Together, these findings showcase what we believe is a novel mechanistic pathway of direct viral clearance in neutrophils dependent on type I interferon signaling through IFIT3 expression.

## Discussion

This study highlights what we believe is a new type I interferon–dependent antiviral function of neutrophils in respiratory infections that curbs COVID-19 immunopathology. Indeed, the loss of these antiviral neutrophils predicted poor clinical outcomes in severely ill COVID-19 patients with ARDS. Further, the identification of this unique cell population provides a novel avenue for cell-directed therapeutics.

Neutrophils have also been identified in other respiratory viral infections, although their roles remain relatively underappreciated. Previous studies focusing on respiratory syncytial virus (RSV) and influenza A virus (IAV) showed that both viruses can be opsonized by the surfactant protein D and phagocytosed by neutrophils (30, 31), and that RSV can undergo transcription in the neutrophils themselves (32), but a role of these cells in direct viral suppression was not ascertained. Further, depletion of neutrophils in vivo upon challenge with IAV led to severe lung pathology and mortality outcomes (33, 34). Relatedly, we recently found that neutrophil populations in cystic fibrosis also undergo transcriptional changes (23), highlighting the potential plasticity of these cells in the lung microenvironment.

Prior studies have suggested that loss of type I interferon activity is detrimental to viral clearance in patients with severe COVID-19 (35, 36), but the relative cell-type contributions to this signaling in the lung are not well known (37). The identification of a type I interferon signature in the A2 neutrophil population provides a discrete pathway by which these cells directly participate in viral clearance. Importantly, by demonstrating loss of virus in A2 neutrophils and then blocking this effect with inhibition of type I interferon signaling, we identified an important mechanism of viral clearance by these innate immune cells. Further, IFIT3 emerged as a potential key regulator of such antiviral functions, as has previously been shown in other immune cells during viral infections (38, 39). Although the mechanism of action for this protein in airway neutrophils remains poorly understood, our work shows that IFIT3 acts as a critical effector in neutrophil-related viral clearance through IRF3-dependent catabolic targeting of viral RNA. Examination of these neutrophils in other respiratory viral infections would provide further insight into how neutrophils may be contributing to antiviral immunity in these disorders. Likewise, manipulation of neutrophilic inflammation in an in vivo model of SARS-CoV-2 infection would provide definitive evidence of the role of these cells in COVID-19. However, while several in vivo models have been developed for SARS-CoV-2 infection, they lack a recapitulation of ALI with neutrophil-dominated inflammation (40, 41). As improved animal models are developed for SARS-CoV-2 ALI, we believe targeting neutrophil phenotypes will be of great interest.

Limited understanding of the immune profile in both systemic and lung compartments in ARDS remains an impediment to the development of appropriate disease-related biomarkers and therapeutics. The present study highlights the largest concomitant analysis of matched blood and airway immune landscapes in COVID-19 patients admitted to the ICU and provides longitudinal analysis of matched matrices using multiparametric flow cytometry. The limited systemic and local modulation of innate immune responses over time suggests that the innate immune landscape and activation present at the onset of ARDS symptoms establish a clinical course of disease. This observation provides an approach to stratify the critically ill patient population, identifying COVID-19 ARDS patients at high risk for death early in their ICU course for close clinical monitoring and early clinical trial recruitment.

To our knowledge, this study provides the first evidence of an antiviral neutrophil subset. This neutrophil subset was also detected in non–COVID-19 ARDS, suggesting a broader role for this subpopulation and warranting more robust immunologic phenotyping in clinical conditions to better discern and inform therapeutics (42), including cell-based therapies (43). We observed A2 neutrophil frequencies as high as 70% of the total immune cells in the lung; therefore, their impact on viral immunity is likely profound. Therapeutic considerations of this cell subset may potentially impact outcomes in both ARDS and viral lung disease.

## Methods

### Sample collection and processing.

Patients were recruited at the Medical Intensive Care Unit (MICU) at the University of Alabama Hospital and at the Brigham and Women’s Hospital. Patients included in the study were intubated, had confirmed SARS-CoV-2^+^ infection, and met the clinical definition of ARDS according to Berlin criteria (44). Patient demographics are provided in Supplemental Table 1. Control ALI mini-bronchoalveolar lavage (mBAL) fluids were obtained from patients presenting with nonviral infectious ALI. Demographics for the ALI control group and healthy blood donors are shown in Supplemental Tables 2 and 3, respectively.

Blood and mBAL fluids were collected from COVID-19 patients at the time of admission to the MICU (*N* = 52) and a subset 1 week afterward (*N* = 28), as described in the Supplemental Methods.

### Flow cytometry and image cytometry.

Multiparametric flow cytometry analysis of whole blood and airway cells was standardized across study visits using the acquisition setting automatic calibration built into the BD software on the BD FACSymphony instrument (BD Biosciences), which provides constant and robust output from the flow cytometer over time. Samples were prestained for 10 minutes on ice in the dark with the Human TruStain FcX Fc blocking solution and the Zombie near-IR reagent (BioLegend), and then stained for surface markers (see Supplemental Table 4 for antibodies). Cells were washed, fixed in Lyse/Fix Phosflow (BD Biosciences), and acquired on a FACSymphony. Analysis and compensation were performed in FlowJo v10.6.2 (BD Biosciences). Image cytometry was performed as previously described (45) (see Supplemental Methods).

### In vitro transmigration.

Purified blood neutrophils were transmigrated in vitro as previously described (24) using ALI/ARDS mBAL supernatant obtained by mechanical dissociation on ice using an 18-G needle and syringe, followed by differential centrifugation at 800*g* and 3,000*g* to obtain the cell- and bacteria-free supernatant (see Supplemental Methods).

### Type I interferon blockade.

A2 neutrophils were transmigrated, as described above, into ALI/ARDS mBAL supernatant supplemented with either 10 μg/mL IgG control (BioLegend) or 10 μg/mL anifrolumab (anti-IFNAR1, Thermo Fisher Scientific; Supplemental Table 4), as previously described (46). Treatment with IgG control antibody or anifrolumab was continued at the same concentration for the first hour of incubation for SARS-CoV-2. Viral infectivity in the presence of these antibodies was tested and did not differ from a control condition with virus alone.

### Extracellular viral clearance assays.

To assess a direct effect on SARS-CoV-2, neutrophil-conditioned media were incubated with 125 foci-forming units (FFU) of SARS-CoV-2 (1:1 by volume) for 30 minutes at 35°C and 5% CO_2_, and then used for foci-forming assays. To determine the presence of an indirect effect, neutrophil-conditioned media were incubated with a monolayer of VERO cells (ATCC) (1:1 dilution in RPMI) for 4 or 24 hours. Then, VERO cells were washed with RPMI and 50 FFU of SARS-CoV-2 was added as detailed in the *Foci-forming assay* section below. Infection rate was assessed by foci-forming assays.

### Intracellular viral clearance assays.

A1 and A2 neutrophils were incubated in plain RPMI with SARS-CoV-2 at an MOI of 1 for 1 hour at 37°C and 5% CO_2_. After incubation, neutrophils were separated from the supernatant after a 10-minute, 500*g* centrifugation. The supernatant was layered on VERO cells for foci-forming assays, while neutrophils were resuspended in DMEM/F-12 media supplemented with 2% 0.1-μm-filtered FBS and incubated for 24 hours at 35°C and 5% CO_2_. Neutrophil viability after 24 hours was assessed at 80%–90% for all conditions. Neutrophils were spun at 500*g* for 10 minutes and the supernatant was used to quantify exocytosed SARS-CoV-2 by foci-forming assay. To determine intracellular viral loads, neutrophils were lysed by freezing at –80°C. Samples were then spun at 500*g* for 10 minutes and the supernatant was used for foci-forming assays.

### Foci-forming assay.

VERO cells and SARS-CoV-2 were prepared as detailed in the Supplemental Methods. Briefly, infection was allowed to proceed for 1 hour on the VERO cells at 35°C. Then, an overlay of Eagle’s MEM with 4% FBS and antibiotics and was added to the inoculum on the cell monolayers, and the infection allowed to proceed for 24 hours. After fixation (see Supplemental Methods), SARS-CoV-2 was detected using a rabbit polyclonal anti-Spike/RBD antibody (40150-T30, SinoBiologicals) with goat anti–rabbit IgG conjugated to horseradish peroxidase (Boster Biological Technology Co.) as secondary antibody. Quantification of foci was determined as detailed in the Supplemental Methods.

### RNA isolation and sequencing.

RNA from noninfected neutrophils was isolated using the Nucleospin RNA kit (Takara).

RNA from infected neutrophils was obtained by use of the RNeasy Plus mini kit (Qiagen) according to manufacturer’s instructions. RNA isolated from in vitro samples was sequenced on the Illumina NextSeq 500 with 75-bp paired-end reads, with a target of 20 million reads per sample. FASTQ files were checked for quality and raw sequencing data were aligned to the human reference genome (GRCh38) using STAR (v2.5.2; https://github.com/alexdobin/STAR/releases) and quantmode was used to generate raw transcript counts. Differential gene expression was determined using DESeq2 (https://bioconductor.org/packages/release/bioc/html/DESeq2.html), while pathway analysis was performed using Ingenuity Pathway Analysis (Qiagen). Data can be accessed in the NCBI Gene Expression Omnibus (GEO) database under accession number GSE228152.

### Single-cell RNA sequencing.

FASTQ files of BAL fluid from 21 severe COVID-19 patients from Bost et al. (26) were downloaded from the European Nucleotide Archive (https://www.ebi.ac.uk/ena/browser/view/PRJNA661032 Accessed June, 2021.). Transcriptomic alignment, barcode demultiplexing, and gene count quantification were done using Cell Ranger (v4.0.0; https://support.10xgenomics.com/single-cell-gene-expression/software/downloads/latest) with the force-cells option set to 10,000. All downstream analyses were done using Scanpy (v1.6.0) (47), Scrublet (v0.2.1), Scran (v1.10.2), UMAP, and MAST (Seurat API), as detailed in the Supplemental Methods and Supplemental Figure 4.

### Viral RNA and viral replication.

SARS-CoV-2 *N1* and *S* RNAs in A2 neutrophils, as well as replication in A1 and A2 neutrophils, were assessed by RT-PCR, with a positive control generated from a mix of clinical SARS-CoV-2^+^ samples (see Supplemental Methods).

### IFIT3 knockdown.

A2 neutrophils were transfected with 10 pmol of *IFIT3* siRNA (s7155, Thermo Fisher Scientific) or of *Silencer* Cy3-labeled Negative Control No. 1 siRNA (Thermo Fisher Scientific) using Lipofectamine RNAiMAX (Thermo Fisher Scientific) in Opti-MEM I reduced serum medium (Thermo Fisher Scientific) as per the manufacturer’s protocol for 3 hours prior to the incubation with SARS-CoV-2. Knockdown efficiency was quantified by RT-PCR (48, 49) (see Supplemental Methods).

### Statistics.

Statistical analyses were performed in JMP Pro 15 (SAS Institute), while graphing was done using Prism v8 (GraphPad) and R. Threshold for A2 frequencies was determined by partitioning analysis followed by ROC curve for mortality (area = 0.64; A2 neutrophil percentage less than 42 defined as “low A2”). Patients included in the study had at least matched blood and BAL fluid at time point 1, while patients who were lacking time point 2 data for reasons other than mortality were excluded from the analysis. All data were analyzed using nonparametric statistics: paired comparisons for each individual between 2 time points were done using Wilcoxon’s matched-pair signed-rank test, nonpaired analyses were performed using Wilcoxon’s rank-sum test, Fisher’s exact test was performed on contingency tables, and correlations were tested using Spearman’s ρ. Data are shown as median and interquartile range. A *P* value of less than 0.05 was considered significant. Details can be found in the figure legends.

### Study approval.

All data and samples were collected in accordance with the University of Alabama at Birmingham’s IRB (COVID Enterprise IRB: IRB-300005127 and IRB-300005209) and at the Brigham and Women’s Hospital (IRB-2008P000495 and IRB-2020P000447). Written consent was obtained prior to participation.

## Author contributions

AG, NBE, and CM conceived the study. DWR, SBP, NSS, and AG collected the samples. CM, TF, MCM, SS, JT, YSV, and MMB performed the experiments. TA, SWR, DWR, and AG curated the clinical outcomes. SDF performed the transcriptional analysis. KSH and SML supervised the viral work. CM and AG wrote the manuscript, which was edited and approved by all authors.

## Supplementary Material

Supplemental data

## Version 1 04/18/2023

In-Press Preview

## Version 2 05/22/2023

Electronic publication
