# Supplementary material for: Interferon-dependent signaling is critical for viral clearance in airway neutrophils
Source: JCI Insight. 2023 May 22;8(10):e167042. doi: 10.1172/jci.insight.167042 (PMC10322684; doi:10.1172/jci.insight.167042)
Supplement: Supplemental data [file jciinsight-8-167042-s072.pdf]

Supplementary Materials for

Interferon-dependent signaling is critical for viral clearance in airway neutrophils

Camilla Margaroli[1,2], Timothy Fram[3+], Nirmal S. Sharma[2,4+], Siddharth B. Patel[1], Jennifer Tipper[5], Sarah W. Robison[1,2], Derek W. Russell[1,2], Seth D. Fortmann[6], Mudassir M. Banday[4], Yixel Soto-Vazquez[1,2], Tarek Abdalla[1,2], Sawanan Saitornuang[7], Matthew C. Madison[1,2], Sixto M. Leal Jr[7], Kevin S. Harrod[5], Nathaniel B. Erdmann[3#], Amit Gaggar[1,2,8,9#*]

Correspondence to: agaggar@uabmc.edu

This file includes:

Supplemental methods
Figs. S1 to S6
Tables S1 to S4

Supplemental methods

Sample collection and processing

Blood was collected in K_2-EDTA tubes by venipuncture, cells and plasma were separated by 400g, 10 minutes, 4°C centrifugation. The cellular fraction was resuspended in PBS-EDTA (2.5mM) to match the collection volume and stained for flow cytometry analyses. mBAL was collected by instillation of three separate 10-ml aliquots into the endotracheal tube via 14 Fr in-line suction catheter and the sample is aspirated back between each aliquot. Collected aspirate was mechanically dissociated on ice using an 18G needle and syringe. Airway immune cells were recovered after an 800g, 10 minutes, 4°C centrifugation, washed with PBS-EDTA (2.5mM) and stained for flow cytometry.

Image cytometry

Airway cells were fixed in BD Lyse/Fix Phosflow (BD Biosciences) and stored at -80 °C until use. Cells were thawed and washed with PBS-EDTA and permeabilized with Perm Buffer I (BD Biosciences) for 15 minutes at room temperature. Staining was performed in Perm Buffer I with DAPI (nuclear stain, 1μM), cholera toxin B-Alexa Fluor 555 (to distinguish neutrophils which contain higher amount of lipid rafts than other immune cells, Thermofisher, 0.1 μg) (15) , as well as antibodies targeting CD63-APC (Biolegend, clone: H5C6, 0.2μL), SARS-CoV-2 nucleocapsid conjugated with FITC (GeneTex, GTX135361, 3μg), IFIT3 (Thermofisher, 1:100, RRID: AB_11153289), and phospho-IRF3 (Thermofisher, 1:100, RRID: AB_2532786). Cells were washed twice with Perm Buffer I and resuspended in PBS-EDTA prior to acquisition. Specimens were acquired on the Amnis Imagestream X Mark II (Luminex Corporation), with 40x magnification and low flow rate/high sensitivity on the INSPIRE software. Brightfield was set on channels 01 and 09, while scattering was set in channel 06. Data were analyzed using the IDEAS software v6.1 (Luminex Corporation).

In vitro transmigration

Neutrophils were isolated from whole blood using the density gradient Polymorphprep (Cosmo Bio USA) following manufacturer protocol (16). Neutrophil activation, purity (99% purity was obtained for every isolation), and viability was assessed using flow cytometry. and allowed to migrate into the chemoattractant LTB_4 (100nM, Sigma) (A1 neutrophils, (16)) or into ALI/ARDS mBAL supernatant diluted 1:1 in plain RPMI (A2 neutrophils). Neutrophils were then collected at 14 hours post-transmigration (all conditions had at least 90% viability), washed, phenotyped by flow cytometry and used for downstream assays. Neutrophil conditioned media was obtained by isolating the supernatant (800g, 10 minutes, 4°C centrifugation) from transmigrated A1 or A2 neutrophils incubated in fresh RPMI for 3 hours post-transmigration.

Vero E6 cell line

Vero E6 cells (ATCC, Cat# C1008) were cultured in 96-well plates until confluence in Eagle's MEM with 4% FBS (Denville Scientific Inc) supplemented with anti-biotic/anti-mycotic (Gibco) containing 100 units/mL of penicillin, 100 µg/mL of streptomycin, and 0.25 µg/mL of Gibco Amphotericin B.

SARS-CoV-2 viral stocks

The original SARS-CoV-2 isolate USA-WA1/2020 was obtained from BEI resources (#NR-52281) and further propagated in Vero E6 cells through 2 more passages to obtain a working stock of virus at sufficient titer. A focus forming assay was used to quantify the titer of viral stocks and virus obtained from subsequent experimental tests.

Foci forming assay

Virus was serially diluted and added to the wells (100µL), and infection allowed to proceed for 1 hour on the VERO cells at 35°C. At the completion of the 1-hour incubation, an overlay of Eagle's MEM with 4% FBS and antibiotics and was added to the inoculum on the cell monolayers such that the final volume was 200 µL per well. The infection was allowed to proceed for 24 hr. The next day, each plate was fixed by submerging the entire plate and contents in 10% formalin/PBS for 24 h. Detection of virus focal units, expressed as foci forming units (FFU) per mL, was performed on fixed 96 well plates. Briefly, plates were rinsed in H_2O, and methanol:hydrogen peroxide (5% H_2O_2 in absolute methanol) added to the wells for 30 min with rocking to quench endogenous peroxidase activity. After quenching, plates were rinsed in H_2O to remove methanol and Blotto (Thermo Scientific; equivalent to 5% non-fat dried milk) was added to the wells as a blocking solution for 1 hour. For primary antibody detection, a SARS-CoV-2 Spike/RBD antibody (Rabbit, Polyclonal, SinoBiologicals) was added to Blotto and incubated on the monolayers for at least 1 hour. Plates were rinsed 5 times with PBS, and further incubated with a secondary antibody of goat anti-rabbit IgG conjugated to horseradish peroxidase (Boster Biological Technology Co.) in Blotto for 1 hour. Plates were rinsed once with 0.05% tween in 1X PBS followed by 5 washes in PBS. Peroxidase activity was detected by use of Impact DAB detection kit (Vector Labs) per manufacturer's instructions. Foci are counted manually from the scanned image of each well or otherwise microscopically imaged and quantitated. For conditions where foci were too dense to count, quantification of FFU was performed by densitometric analysis using Image Studio v5.2.5 on scanned wells (LI-COR Biosciences). All steps involved in handling viable virus were performed in a BSL-3 facility.

Single-Cell RNA-sequencing

The reference transcriptome was GRCh38 and was downloaded from 10X Genomics. All downstream analyses were done using Scanpy (version 1.6.0), a Python-based suite of packages for scRNA-seq analysis. For quality control filtering, cells were removed that contained <500 reads and <500 genes. Additionally, mitochondrial and ribosomal gene percentage cutoffs of >40% and >50%, respectively, were used to further eliminate low quality cells. Lastly, Scrublet (version 0.2.1) was used to remove potential doublets. After quality control filtering, the 21 samples were concatenated and 48,582 cells were recovered in total. Normalization was performed using Scran (version 1.10.2), and Scanpy was then used to perform complete cell cycle regression using the cell cycle genes identified by Tirosh and colleagues (19). Scanpy was used to select the top 1,500 highly variable genes, which were then used to calculate the top 13 principal components (PCs). Batch correction was performed using Harmony with the Scanpy external application programming interface (API; **Figure S4**). One categorical covariate was used for Harmony integration designating individual patient samples. Final dimensionality reduction was done using uniform manifold approximation and projection (UMAP) with default settings. Clustering was performed using the Leiden algorithm with a resolution of 0.3 followed by subclustering, and 8 clusters were identified. The assignment of cluster identities was guided by the expression of lineage-specific marker genes (**Figure S4**). Differential gene expression analysis was performed on normalized expression data using MAST (Seurat API).

Viral RNA and viral replication

The following primer sequences were used: Replication - Forward = CTCTTGTAGATCTGTTCTCTAAACGAAC, Reverse = GGTCCACCAAACGTAATGCG;

N1 – Forward = GACCCCAAAATCAGCGAAAT,
Reverse=TCTGGTTACTGCCAGTTGAATCTG;
S (provided by Matt Frieman, Emory University, Atlanta, GA)–
Forward=CTCTTGTAGATCTGTTCTCTAAACGAAC,
Reverse = GGTCCACCAAACGTAATGCG;
RP – Forward = AGATTTGGACCTGCGAGCG, Reverse =
GAGCGGCTGTCTCCACAAGT. Primers were used at a final concentration of 200nM with Power SYBR® Green RNA-to-CT™ 1-Step Kit (Thermofisher). Reverse transcription was performed at 48°C for 30 minutes, followed by activation at 95°C for 10 minutes. Amplification was carried out over 40 cycles as follow: denaturation (95°C, 15 seconds), annealing/extension (58°C, 1 minute). The melting curve was measured as follow: denaturing (95°C, 15 seconds), annealing (60°C, 15 seconds), denaturing (95°C, 15 seconds).

<u>IFIT3 knockdown</u>

Knockdown efficiency was quantified by RT-PCR using the following primers: *IFIT3* - Forward = GAACATGCTGACCAAGCAGA; Reverse = CAGTTGTGTCCACCCTTCCT) (20,21); *ACTB* – Forward = AAAGACCTGTACGCCAACAC, Reverse = GTCATACTCCTGCTTGCTGAT. Primers were used at a final concentration of 200nM with Power SYBR® Green RNA-to-CT™ 1-Step Kit (Thermofisher). Reverse transcription was performed at 48°C for 30 minutes, followed by activation at 95°C for 10 minutes. Amplification was carried out over 40 cycles as follow: denaturation (95°C, 30 seconds), annealing (58°C, 1 minute), extension (72°C, 30 seconds). The melting curve was measured as follow: denaturing (95°C, 15 seconds), annealing (60°C, 15 seconds), denaturing (95°C, 15 seconds).

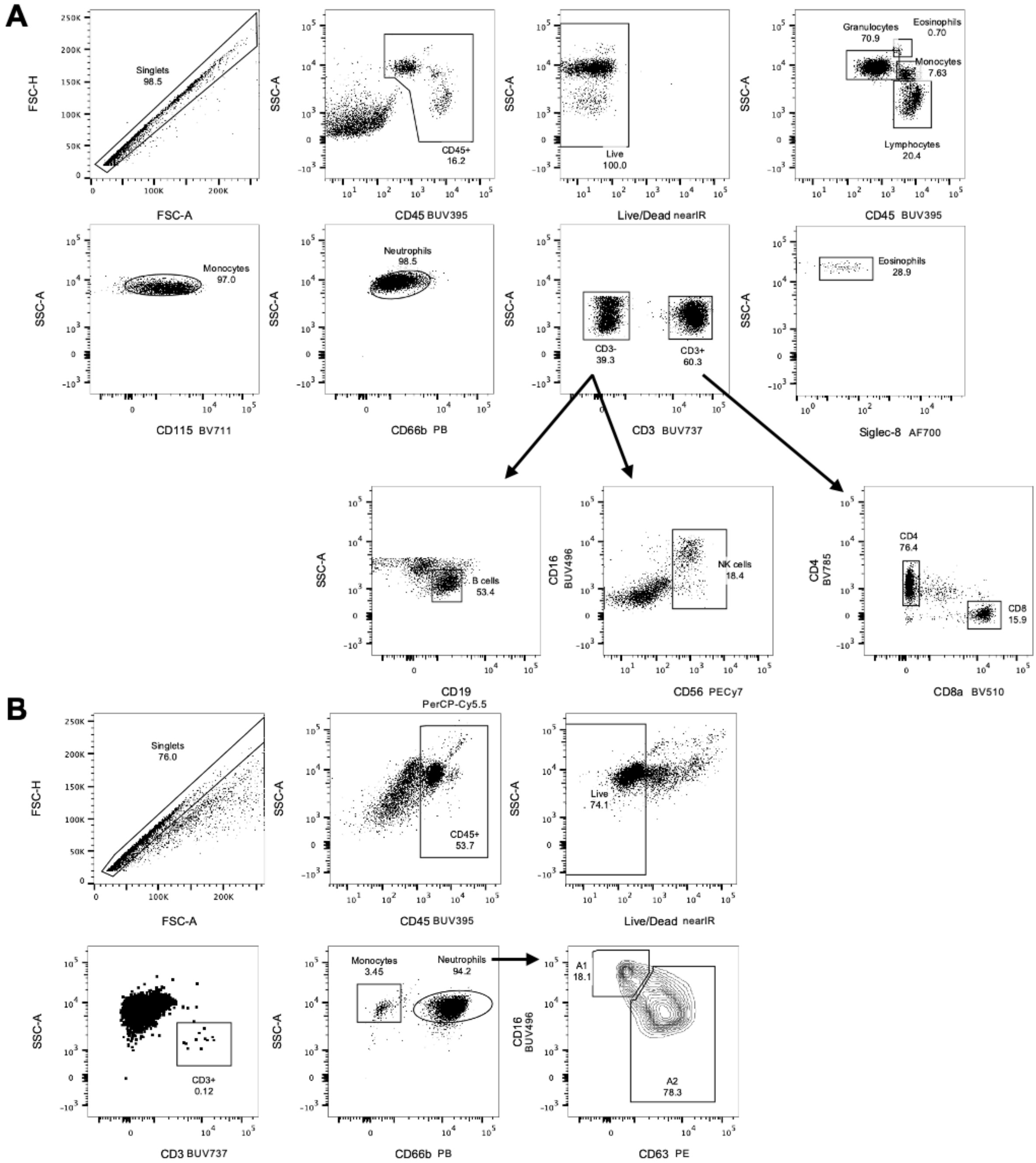

Fig. S1. Flow cytometry gating strategy. Gating strategy for blood and airway immune cells. Blood **(A)** and airway **(B)** immune cells frequencies were quantified out of live and CD45+ cells.

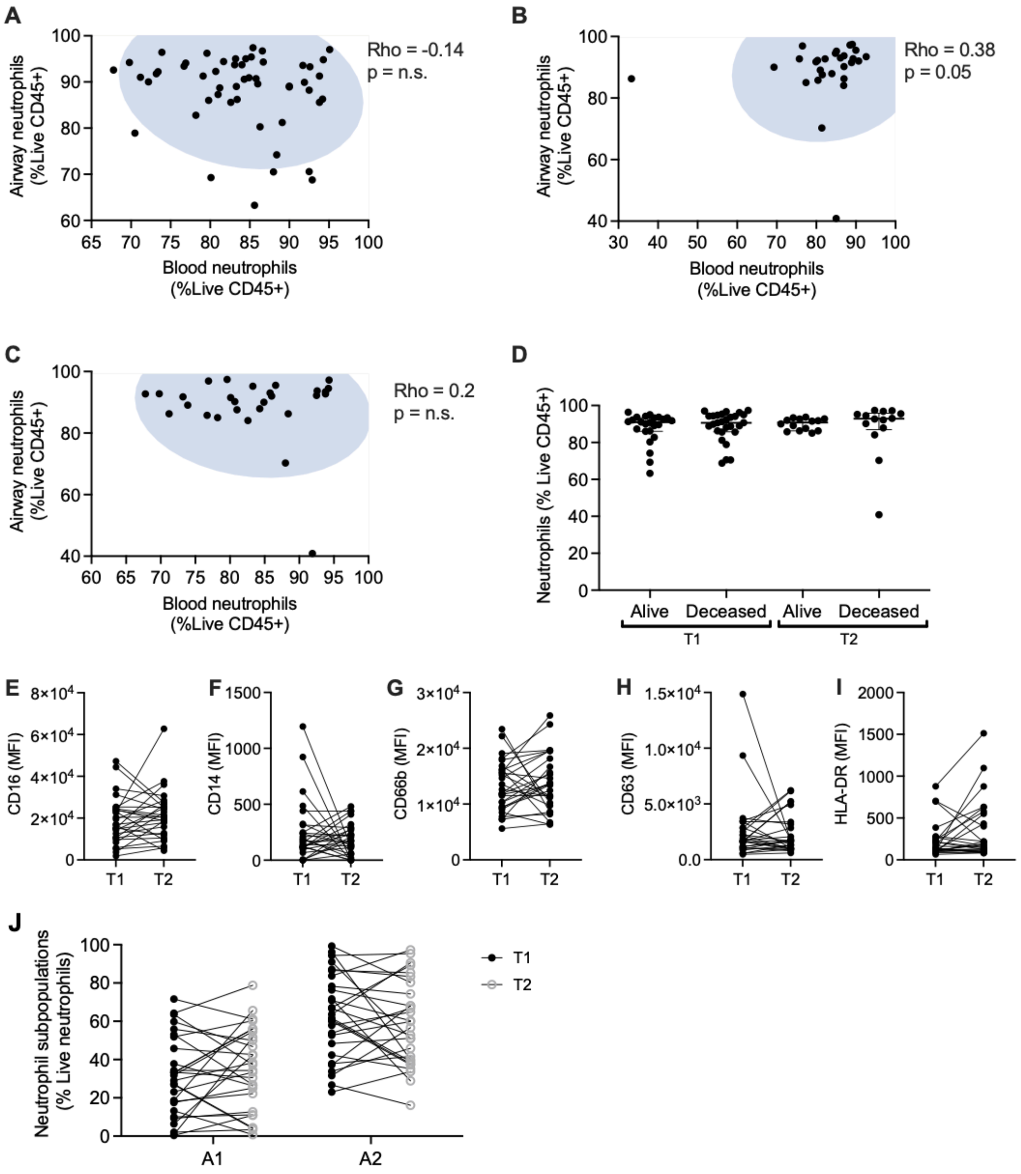

Fig. S2. Airway neutrophil frequencies over 7 days of ICU admission. Airway neutrophil frequencies (see figure S1 for gating strategy) were quantified by flow cytometry. Blood and airway neutrophil frequencies did not correlate at either time point 1 **(A)**, at time point 2 **(B)**, or longitudinally between blood at T1 and airways at T2 **(C)**. **(D)** No differences were observed in airway neutrophil frequencies upon mortality state or longitudinally within each group. Each point represents a single patient at time of ICU admission (T1, n=52 – 28 alive, 24 deceased), and 5-7 days later (T2, n=28 – 14 alive, 14 deceased). **(E-I)** Likewise, activation profiles of airway neutrophils did not show differences between time points 1 and 2 in patients having measures for both time points (n = 28, MFI: median fluorescence intensity). **(K)** A1 and A2 neutrophil frequencies at T1 and T2. Correlations were analyzed by Spearman's Rho, blue areas show 90% confidence interval by bivariate normal density ellipse. Statistical analysis was performed using Mann-Whitney's test to compare live and deceased patients within each time point, and the Wilcoxon matched-pair signed rank's test for longitudinal comparison. Data are shown as median and interquartile range, all p-values were above the 0.05 threshold of significance.

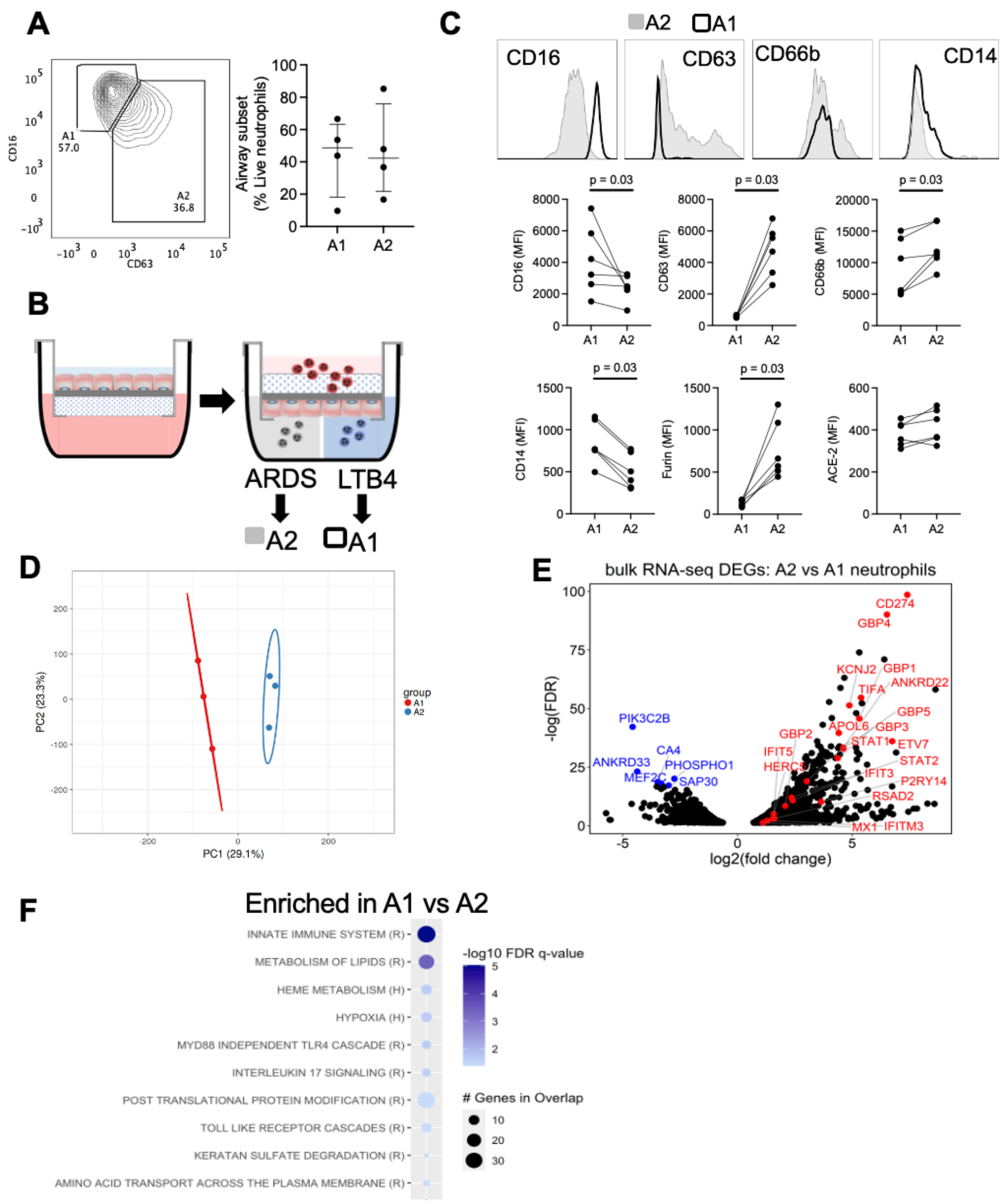

Fig. S3. Neutrophil-SARS-CoV-2 interactions in vitro. (A) Flow cytometry phenotyping of airway neutrophils from Gram negative sepsis ARDS patients (n = 4). **(B)** Schematics of the in vitro transmigration model used to generate A1 (LTB_4) and A2 (ARDS supernatant) neutrophils. Details can be found in the methods. **(C)** Airway neutrophil phenotyping by flow cytometry upon in vitro generation of A1 and A2 subsets (n = 6). **(D)** PCA analysis of RNAseq data from in vitro A1 and A2 neutrophils. **(E)** Differentially expressed genes in A2 neutrophils compared to A1. **(F)** Pathway analysis for genes enriched in A1 neutrophils generated in vitro (n=3 donors). Data are shown as median and interquartile range, Statistical analysis was performed using Wilcoxon matched-pair signed rank's test.

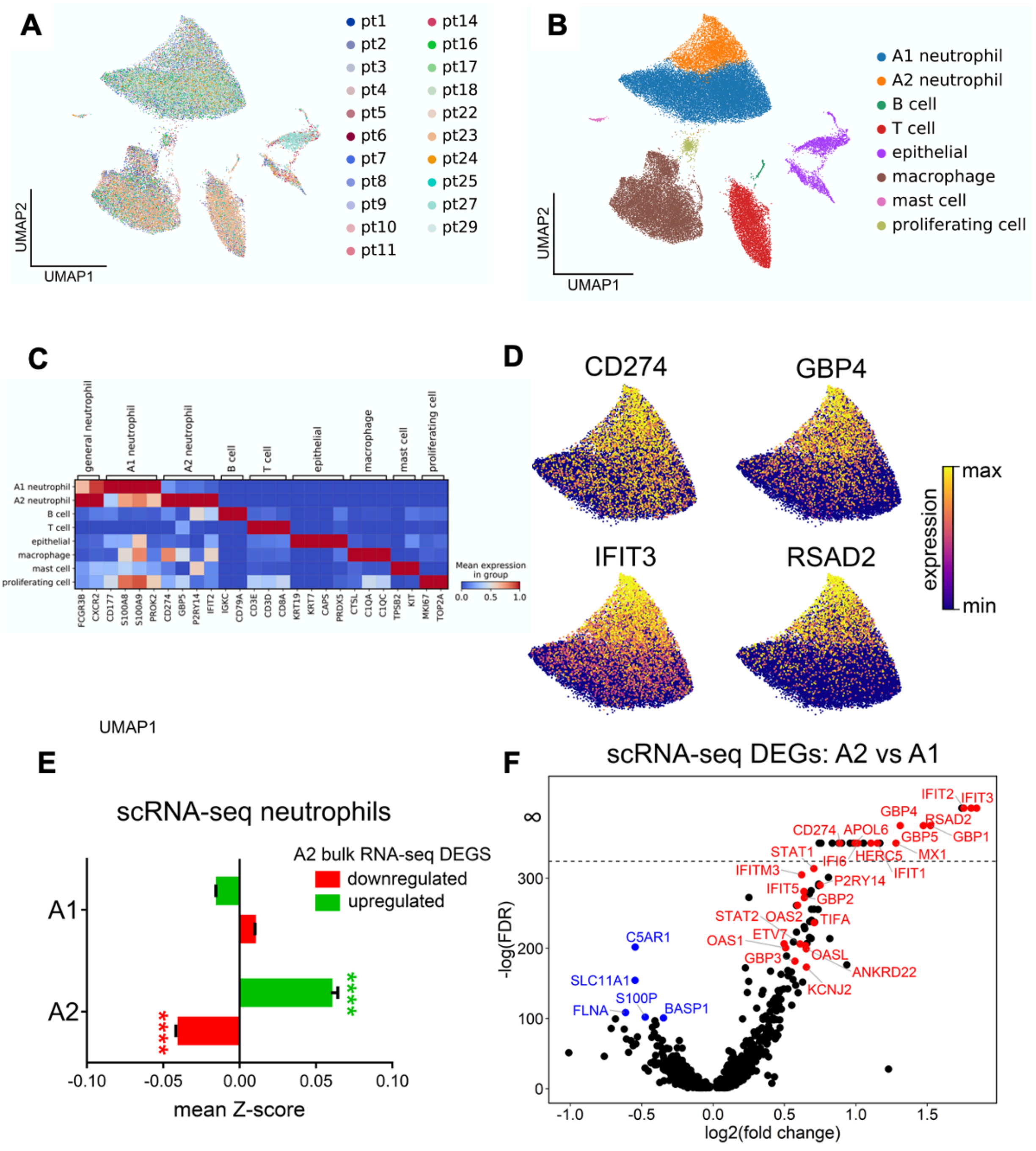

Fig. S4. Transcriptomic identification of A1 and A2 neutrophils in COVID-19 bronchioalveolar lavage. (A) Single-cell RNA-seq of bronchoalveolar lavage in 21 severe COVID-19 patients. **(B)** Single-cell RNA-seq (scRNA-seq) of neutrophils (25,664 cells) from bronchioalveolar lavage (BAL) from 21 severe COVID-19 patients. 20,660 A1 neutrophils and 5,064 A2 neutrophils. **(C)** Core gene expression for population identification in scRNA-seq. **(D)** scRNA-seq expression of A2 neutrophil marker genes. **(E)** Expression of A2 differentially expressed genes (DEGs) from in vitro bulk RNA-seq in A1 and A2 neutrophils from BAL scRNA-seq. **(F)** Volcano plot of DEGs in A2 vs A1 neutrophils from BAL scRNA-seq. 1,402 DEGs: 596 upregulated, 806 downregulated.

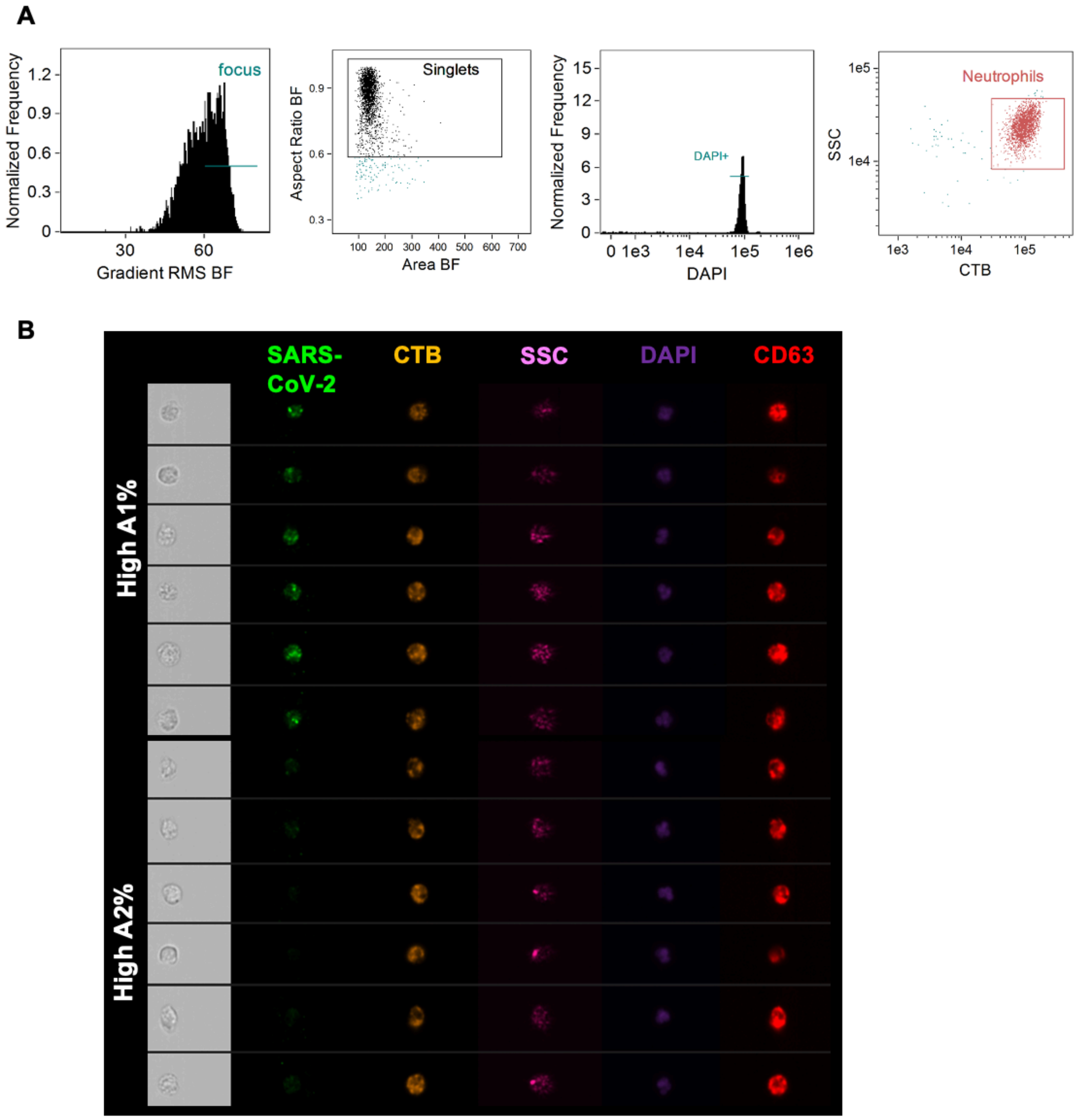

Fig. S5. ImageStream analysis of airway immune cells. (A) From left to right: gradient RMS brightfield (BF) gating was used to obtain focused events; then single cells were gated using Aspect Ratio BF vs. Area BF; Live cells (diploid) were obtained using DAPI staining; airway neutrophils were gated based upon higher level of cholera toxin B (CTB) staining and side scatter (SSC) (Laval et al., 2013), and validated by images collected during the acquisition. (**B)** BF, SARS-CoV-2 nucleocapsid (green), CTB (orange), SSC (pink), DAPI (purple), and CD63 (red) expression were measured. Patients with high A1% (n = 6) showed increased staining for SARS-CoV-2 nucleocapsid compared to patients with high A2 frequencies (n = 6).

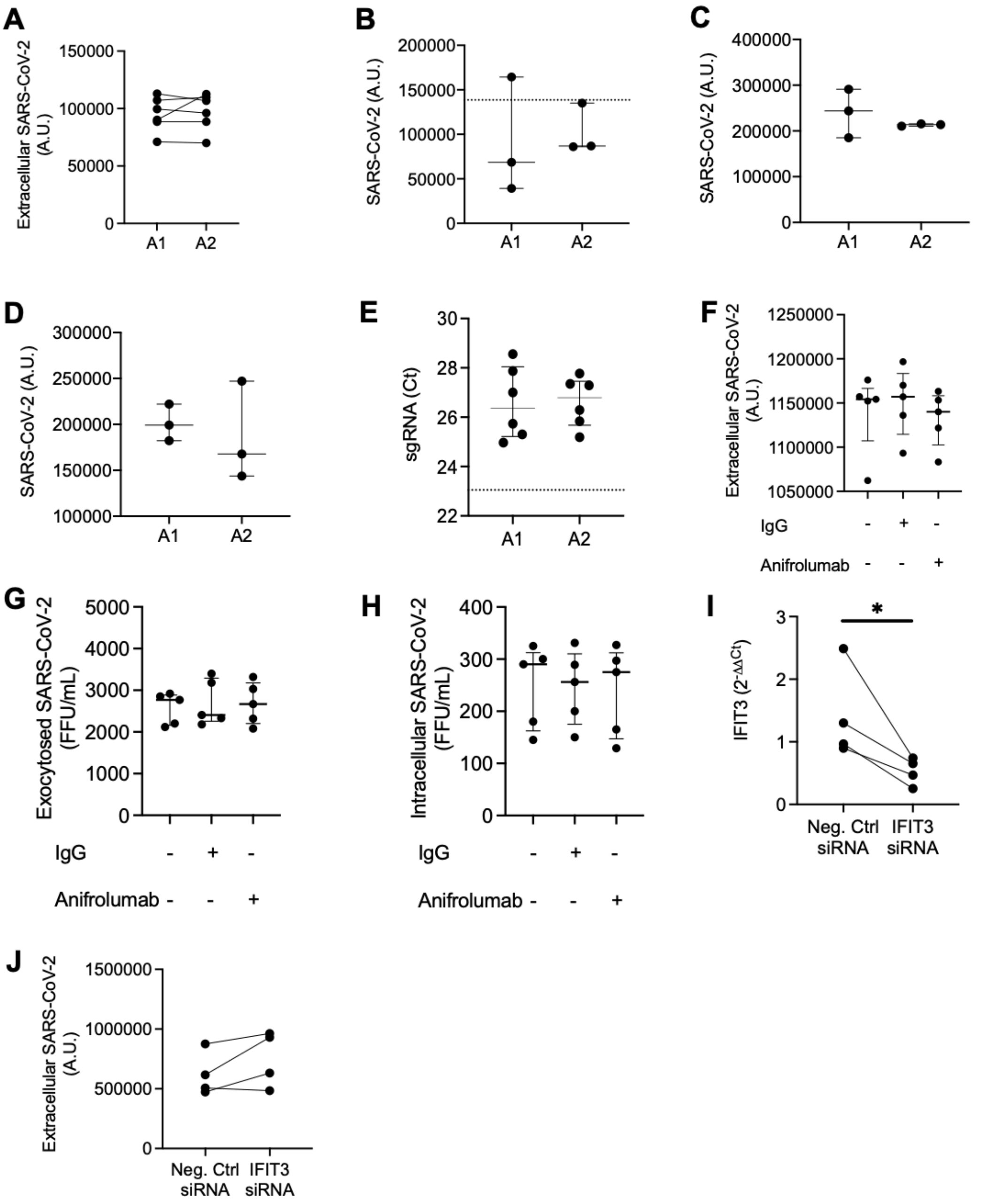

Fig. S6. Neutrophil-SARS-CoV-2 interactions in vitro. (A) A1 and A2 neutrophils incubated with SARS-CoV-2 (MOI = 1) showed similar rate of viral uptake (n=6 neutrophil donors). **(B)** Neutrophil conditioned media did not affect SARS-CoV-2 infectivity directly or viral resistance of VERO cells after 4hr **(C)** or 24hr **(D)** incubation (n = 3 neutrophil donors). **(E)** Subgenomic RNA (sgRNA) detection by RT-PCR of SARS-CoV-2 in A1 and A2 neutrophils (dotted line represents positive control, n = 6 neutrophil donors). **(F-H)** SARS-CoV-2 quantification upon A1 neutrophils treated with IgG control or Anifrolumab for viral uptake (extracellular), virus exocytosis, and intracellular virus, respectively. **(I)** RT-PCR of *IFIT3* upon knockdown in A2 neutrophils (n = 4 neutrophil donors). **(J)** Viral uptake by A2 neutrophils upon *IFIT3* knockdown. Statistical analysis was performed using the Wilcoxon matched-pair signed rank's test or Friedman's test (panels F-H). Data are shown as median and interquartile range (A.U. = arbitrary units), * p<0.05.

Table S1.

	Time point 1 (N=52)	Time point 2 (N=28)
**DEMOGRAPHICS**		
Age in years	64 (48.8-71)	63.5 (48.8-71)
Race (Caucasian: African American: Asian :Hispanic)	27:22:2:1	13:13:2:0
Gender (Male n, %)	34 (65.4%)	15 (53.6%)
**COMORBIDITIES**		
Diabetes Mellitus (n, %)	24 (46.2%)	
CV Disease (n, %)	44 (84.6%)	
Chronic Lung Disease (n, %)	14 (26.9%)	
Chronic Kidney Disease (n, %)	14 (26.9%)	
**INTERVENTIONS**		
Remdesivir (n, %)	44 (84.6%)	
Corticosteroids (n, %)	46 (88.5%)	
Nebulized epoprostenol (n, %)	38 (73.1%)	
ECMO (n, %)	2 (3.8%)	
**ICU ADMISSION LABS**		
BMI (kg/m^2)	33.6 (28.9-38.7)	n/a
% Neutrophils	86.5 (82-92.3)	n/a
% Lymphocytes	6 (2-12)	n/a
Procalcitonin (ng/mL)	0.26 (0.1-1.2)	n/a
Ferritin (ng/mL)	650.5 (235.8-1171), n/a =4	n/a
CRP (mg/L)	146.6 (86.8-185.7), n/a =5	n/a
D-Dimer (ng/mL)	777 (426.5-1908), n/a =3	n/a
PT (sec)	14.4 (13.2-15.3), n/a =2	n/a

LDH (U/L)	459 (329-580.8), n/a =6	n/a
Creatinine (mg/dL)	1.45 (0.9-2.7)	n/a
**PHYSIOLOGY**		
PaO2:FiO2 ratio	135 (110-182)	142 (99.3-164.8)
PEEP	10 (8-12)	10 (8-11)
Tidal volume (cc/kg)	5.8 (5.6-6.2)	5.8 (5.4-6.3)
APACHE II Score	24.5 (18.3-28.8)	n/a
SOFA Score	11 (9-13)	11 (9-14)
**30-DAY OUTCOME**		
Hospital days	23 (16-30)	
ICU days	18 (11.3-28)	
Intubation days	16 (9-25.8)	
Death (n, %)	28 (53.8%)	

Table S1. Summary of patient demographics. Data are shown as median and interquartile range, or n and %. Abbreviations: PEEP: positive end-expiratory pressure, FiO2: fraction of inspired oxygen, ECMO: extracorporeal membrane oxygenation; BMI: body mass index; WBC: white blood cells; CRP: C-reactive protein; PT: prothrombin time; LDH: lactic acid dehydrogenase; PaO2: partial pressure arterial oxygen; APACHE: acute physiology and chronic health evaluation; SOFA: sequential organ failure assessment; ICU: intensive care unit.

Table S2.

DIAGNOSIS	GENDER	AGE (YEARS)	RACE	PAO2:FIO2 RATIO
Non viral infectious ALI	M	62	Asian	77
Non viral infectious ALI	F	66	African American	272
Non viral infectious ALI	M	47	Caucasian	87
Non viral infectious ALI	F	61	Caucasian	145.7

Table S2. Summary of acute lung injury cohort demographics. Abbreviations: ALI: Acute Lung Injury; FiO2: fraction of inspired oxygen; PaO2: partial pressure arterial oxygen; M: male; F: female.

Table S3.

DIAGNOSIS	GENDER	AGE (YEARS)	RACE
Healthy	F	30	Caucasian
Healthy	M	46	Asian
Healthy	F	42	Caucasian
Healthy	M	41	Caucasian
Healthy	M	45	African American
Healthy	F	63	Hispanic

Table S3. Summary of healthy donors cohort demographics. Abbreviations: M: male; F: female

Table S4

Antibody	SOURCE	IDENTIFIER
ACE-2	Bioss	Cat# Bs1004R-PE<br>RRID :AB_11075350
CD14	BD Biosciences	Cat# 612902<br>RRID : AB_2870189
CD16	BD Biosciences	Cat# 612944<br>Clone : 3G8
CD19	Biolegend	Cat# 302230<br>RRID : AB_2073119
CD115	Biolegend	Cat# 347318<br>RRID : AB_2687278
CD126	Biolegend	Cat# 352810<br>RRID : AB_2562717
CD182 (CXCR2)	BD Biosciences	Cat# 744197<br>RRID : AB_2742049
CD27	Thermofisher	Cat# 61-0279-42<br>RRID : AB_2574546
CD206	BD Biosciences	Cat# 741641<br>RRID : AB_2871041
CD3	BD Biosciences	Cat# 612750<br>RRID : AB_2870081
CD4	Biolegend	Cat# 317442<br>RRID : AB_2563242
CD45	BD Biosciences	Cat# 563792<br>RRID : AB_2869519
CD56	Biolegend	Cat# 362510<br>RRID : AB_2563927
CD62L	Biolegend	Cat# 304814<br>RRID : AB_493582
CD63	Biolegend	Cat# 353004<br>RRID :AB_10897809
CD66b	Biolegend	Cat# 305112<br>RRID : AB_2563294
CD8a	Biolegend	Cat# 300934<br>RRID : AB_2814115
EGFR	Biolegend	Cat# 352906<br>RRID :AB_11150410
EpCAM	BD Biosciences	Cat# 748381<br>RRID : AB_2872800
Furin	R&D Systems	Cat# IC1503R<br>Clone : 222722
HLA-DR	Biolegend	Cat# 307640<br>RRID : AB_2561913
IFNAR1	Thermofisher	Cat# MA5-32006<br>RRID : AB_2809300
NE	R&D Systems	Cat# IC91671G<br>Clone : 950317
PD-1	Biolegend	Cat# 329950<br>RRID : AB_2566362
SARS-CoV-2 nucleocapsid	GeneTex	Cat# GTX135361<br>RRID : AB_2887484

Siglec-8	R&D Systems	Cat# FAB7975N<br>Clone : 837535
IFIT3	Thermofisher	Cat# PA5-22230<br>RRID: AB_11153289
Phospho-IRF3	Thermofisher	Cat# 720012<br>RRID: AB_2532786

Table S4. List of antibodies used for flow and image cytometry.
